# A Novel Modification of Copper (II) Phthalocyanine Particles towards Electrophoretic Displays

**DOI:** 10.3390/mi13060880

**Published:** 2022-05-31

**Authors:** Yao Wang, Zhi Zhang, Qun Chen, Caihong Ye, Jiahao Zhang, Qingguo Gao, Liming Liu, Jianjun Yang, Xinjian Pan, Yu Miao, Feng Chi, Mingliang Jin

**Affiliations:** 1 School of Electronics and Information, University of Electronic Science and Technology of China, Zhongshan Institute, Zhongshan 528402, China; wang.yao@m.scnu.edu.cn (Y.W.); chenqunscnu@163.com (Q.C.); 13430093058@163.com (C.Y.); zjh1401507126@126.com (J.Z.); gqgemw@163.com (Q.G.); liulmxps@126.com (L.L.); sdyman@uestc.edu.cn (J.Y.); xinjpan@163.com (X.P.); myseeking@126.com (Y.M.); chifeng@semi.ac.cn (F.C.); 2 South China Academy of Advanced Optoelectronics, South China Normal University, Guangzhou 510006, China; jinml@scnu.edu.cn

**Keywords:** electrophoretic display (EPD), electronic ink (E-ink), electrophoretic particles, mono ionic liquid, charge control agent (CCA)

## Abstract

Electrophoretic display (EPD) is a popular display technology in recent years. The core of the EPD is electrophoretic particles, and its Zeta potential has an important impact on EPDs. In this work, a method using pyrrolidine mono ionic liquid was proposed to improve the Zeta potential of electrophoretic particles: Copper (II) phthalocyanine pigment was modified with mono ionic liquid 1-Butyl-1-methylpyrrolidinium bromide. The characterization results show that the mono ionic liquid had been successfully coated on pigment particles. At the same time, the dispersion and stability of particles were improved. The modified Copper (II) phthalocyanine pigment could be stably dispersed in tetrachloroethylene for more than 20 days. The Zeta potential increased from 32.42 mV to 49.91 mV, increasing by 53.95%. Finally, the prepared blue electrophoretic particles were compounded with white titanium dioxide to prepare blue and white dual-color electrophoretic dispersion, and then an EPD cell was designed to test its performance. The results show that the prepared electrophoretic dispersion can realize reversible reciprocating motion. Therefore, because of the unique structure and properties of pyrrolidine mono ionic liquids, the blue nanoparticles prepared with pyrrolidine ionic liquids as charge control agents in this study can be used as excellent candidate materials for EPD.

## 1. Introduction

Electronic paper is a very promising optoelectronic device [1,2,3,4,5], in which electrophoretic display (EPD) is a reflective display technology based on the principle of electrophoresis. The color alternating display is realized by the movement of charged nanoparticles between electrodes under the action of an electric field [6,7,8,9]. It was first proposed by Ota [10] in 1970. As a new display technology, EPD has the advantages of low power consumption, wide viewing angle, and high reliability [11,12,13,14,15,16,17]. Black and white EPD is a mature commercial product [18,19], but color EPD has better application prospects in the market [20]. At present, it mainly depends on color filter film technology to achieve color display, manufacturing color EPD still has certain challenges [21,22]. The composition of the electrophoretic display ink (E-ink) in EPDs includes electrophoretic particles, charge control agent (CCA), dispersant, and dispersion medium [23]. The selection of electrophoretic particles is important because it determines the image quality and contrast [24,25]. Therefore, many studies have focused on the modification of electrophoretic particles [26]. General inorganic particles have good optical properties, but their density is too high, they are easy to agglomerate after a few days, and their suspension stability is poor, so it is not easy to achieve the effect of bistability. Meanwhile, the surface modification process of inorganic particles is complex, but electrophoretic particles based on organic pigment have the advantages of simple surface modification, good chemical durability, and various varieties, which are more suitable to produce EPDs [27,28,29]. Copper (II) phthalocyanine is an organic pigment widely used in dyes and inks. It has the advantages of small particles, low surface polarity, and bright blue. It has excellent light resistance, heat resistance, acid resistance, alkali resistance, and chemical resistance. It is suitable for electrophoretic particles [30,31,32,33].

There are many methods for modifying electrophoretic particles. Fang et al. [34] used the method of the surface coating to prepare negatively charged green composite hollow nanoparticles for EPDs, with a zeta potential of −80 mV. Eshkalak et al. [35] used surface adsorption to prepare two blue electrophoretic particles with a zeta potential of 41.60 mV and 48.00 mV, respectively. Hu et al. [36] used the method of the surface coating to prepare electrophoretic particles with a zeta potential of 4.13 mV. The key to modification is to improve the surface potential and stability of electrophoretic particles in non-polar media. In addition to the methods of surface coating and surface grafting, the purpose can also be achieved by directly adsorbing CCAs. Therefore, the role of the CCA in EPDs is important. Compared with polar media, it is much more difficult to generate charge in non-polar media [37]. Therefore, CCA must have the ability to ionize in organic media. In recent years, it has been reported that pigment particles were modified with anionic surfactants (Sodium dodecyl sulfate [38], Sodium oleate [39,40,41]), cationic surfactants (Cetyltrimethylammonium bromide [42]), non-ionic surfactants (Polyvinylpyrrolidone [43], Span [44,45,46]), or hyperdispersants [47,48] to improve the dispersion and surface charge of pigment particles. Ionic liquids are salt-containing compounds composed of organic cations and inorganic or organic anions. They are “green” chemical reagents with good chemical and thermal stability, low volatility, and high conductivity [49,50,51]. It can be used as a reaction medium to synthesize a variety of nanomaterials [52,53,54,55], or in the fields of medicine and pharmacy [56,57,58], adsorption [59], polymer modifier [60,61], supercapacitor [62], and so on. There are relatively few studies on the modification of electrophoretic particles by ionic liquids as CCAs. The structure determines the properties, and different ionic liquids will have different effects. The cation type of ionic liquid, the length of the cation side chain, and the radius of the anion will all have a certain influence on the ionization degree of ionic liquid in a non-polar medium. Therefore, different ionic liquids are used as CCAs to modify electrophoretic particles, and the effect will be different.

In this study, we used pyrrolidine mono ionic liquid to modify the surface of Copper (II) phthalocyanine pigment and prepared blue electrophoretic particles with positive charges. In addition, due to the existence of the pyrrolidine mono ionic liquid, the Zeta potential of Copper (II) phthalocyanine was increased. The increase of the charge of pigment particles indicates that the stability and electrophoretic properties of pigment have been improved. Finally, the self-made blue electrophoretic particles and white negatively charged titanium dioxide particles were mixed and dispersed in Tetrachloroethylene to prepare the blue and white E-ink, which was poured into the EPD cell, and an obvious contrast image was obtained.

## 2. Materials and Methods

### 2.1. Materials and Reagents

Copper (II) phthalocyanine (CP) (99%), 1-Butyl-1-methylpyrrolidinium bromide (99.0%), Potassium bromide, and tetrachloroethylene (TCE) (99%, Water ≤ 50 ppm) were all purchased from Macklin. Anhydrous ethanol was purchased from Tianjin Da Mao. Sorbitan monooleate (Span 80), and titanium dioxide (98%) were purchased from Aladdin. All reagents could be used without further purification. Ultrapure water was used in the whole experiment. Two pieces of 3 cm × 6 cm Indium-Tin oxide (ITO) transparent conductive glasses (90 ± 20 Ω/sq), which purchased from Shenzhen Laibao High Tech Co., Ltd., Shenzhen, China.

### 2.2. Surface Modification of CP with Mono Ionic Liquids

**Figure 1:**
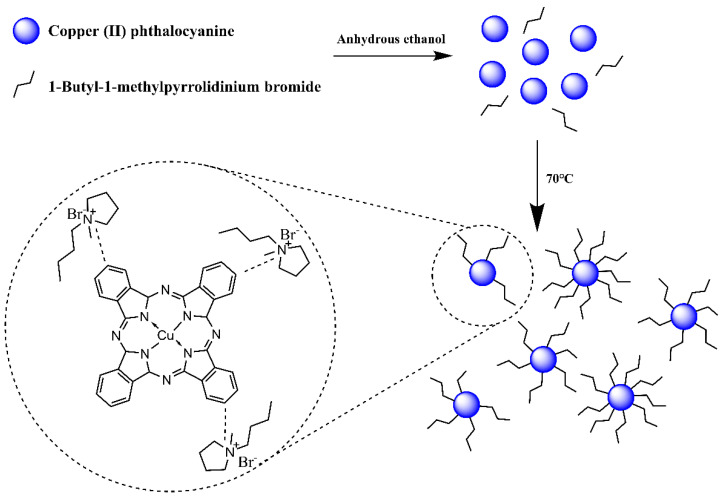
Schematic diagram of modification process and principle of Copper (II) phthalocyanine (CP) use 1-Butyl-1-methylpyrrolidinium bromide (IL), and the structure of the CP modified with IL (CP-IL).

50 mL of anhydrous ethanol and 0.1 g of 1-Butyl-1-methylpyrrolidinium bromide (IL) were added to a 100 mL flask containing 0.5 g CP and vibrated with ultrasound for 10 min. The mixture was stirred at 50 °C for 1.5 h, and then the ethanol was removed at 70 °C with a vacuum dryer. CP modified with 1-Butyl-1-methylpyrrolidinium bromide (CP-IL) was obtained. The modification process of Copper (II) phthalocyanine is shown in the figure (Figure 1).

### 2.3. Preparation of Electrophoretic Dispersion

2 mg CP-IL and 0.5 mg Span 80 were added to 10 mL TCE. The blue electrophoretic dispersion was obtained by ultrasonic dispersion for 30 min.

2 mg CP-IL, 1 mg titanium dioxide, and 0.1 mg Span 80 were added to 10 mL TCE. The blue and white dual-color electrophoretic dispersion was obtained by ultrasonic dispersion for 30 min.

### 2.4. EPD Cell and EPD Experimental Platform

**Figure 2:**
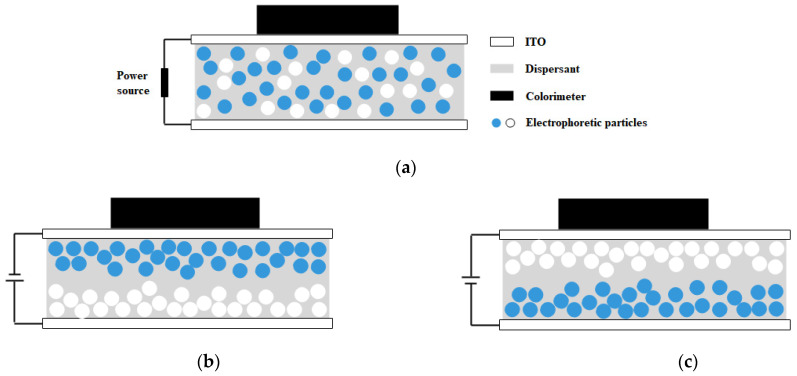
Schematic diagram of a blue and white dual-color electrophoretic display (EPD) cell (**a**). Schematic diagram of the blue and white dual-color EPD cell with positive charges on the lower plate (**b**). Schematic diagram of the blue and white dual-color EPD cell with positive charges on the upper plate (**c**).

AB glue was used to bond two ITO glasses with conductive surfaces to conductive surfaces, leaving a 1 mm high gap in the middle. The blue and white dual-color EPD cell was obtained by injecting the blue and white electrophoretic dispersion into the cell with a syringe (Figure 2a). We can see that when no voltage was applied, the blue and white electrophoretic particles were randomly distributed. When a voltage was applied, the blue and white electrophoretic particles would move and display different colors (Figure 2b,c). When a positive voltage was applied to the lower plate, the negatively charged white electrophoretic particles move down and the positively charged blue electrophoretic particles move up to display dark blue; Instead, white was displayed.

**Figure 3:**
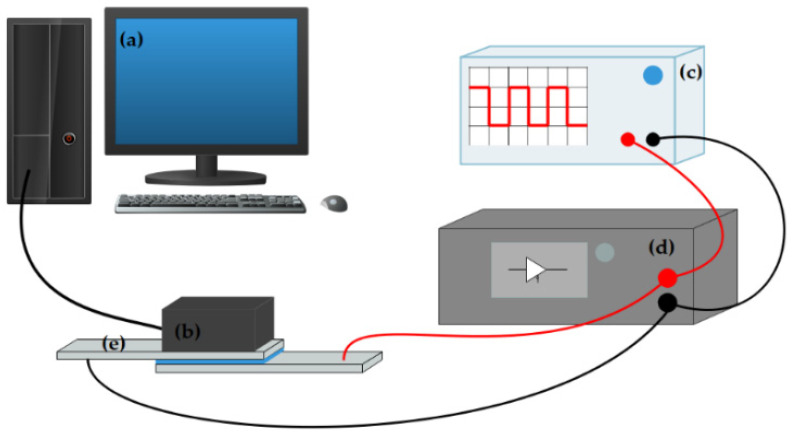
An experimental platform of the electrophoretic display (EPD) cell. The experimental platform consists of a computer (**a**), a colorimeter (**b**), a function generator (**c**), a voltage amplifier (**d**), and an EPD cell (**e**).

The performance of the EPD cell could be determined by testing the Commission International de L’Eclairage (CIE) Yxy chromaticity diagram. Therefore, an experimental platform was developed to test these parameters (Figure 3).

### 2.5. Instruments and Characterization

Scanning electron microscopy (SEM) and energy dispersive spectroscopy (EDS) (Sigma 300, Smartedx, ZEISS, Jena, Germany) were used to characterize the morphology, element composition, and size of CP and CP-IL.

CP, CP-IL, IL, and potassium bromide powder were dried 5 h in a 70 °C vacuum oven to prepare potassium bromide tablets respectively. The functional groups and chemical bonds of the samples were characterized by Fourier transform infrared spectroscopy (FT-IR) (irafficity, Shimadzu, Japan) in the wavelength range of 400–4000 cm^−1^ at 25 °C.

An appropriate amount of CP-IL and CP was dispersed into tetrachloroethylene, ultrasonic for 30 min, and Zeta potential and particle size were measured by Zeta potential and particle size analyzer (Nanobrook 90 plus pals, Brookhaven, NY, USA) at 25 °C.

CP, CP-IL, and IL were dried in an oven at 70 °C for 3 h. Under nitrogen atmosphere, samples were performed at a heating rate of 5 °C/min from 30 °C to 800 °C using a thermal analyzer (STA 449f3, Netzsch, Germany).

An experimental platform was developed to test the the blue and white dual-color EPD cell. It was composed of a driving system and a testing system. The driving system was com-posed of a function generator (AFG3022C, Tektronix, Beaverton, OR, USA), and a voltage amplifier (ATA-2022H, Agitek, Xi’an, China), which was used to generate driving waveforms. The testing system was composed of a computer (H430, Lenovo, Beijing, China), and a colorimeter (Arges-45, Admesy, Ittervoort, The Netherlands), which was used to record the CIE Yxy chromaticity diagram of the EPD cell.

## 3. Results and Discussion

### 3.1. Energy Dispersive Spectroscopy (EDS) and Scanning Electron Microscope (SEM)

**Figure 4:**
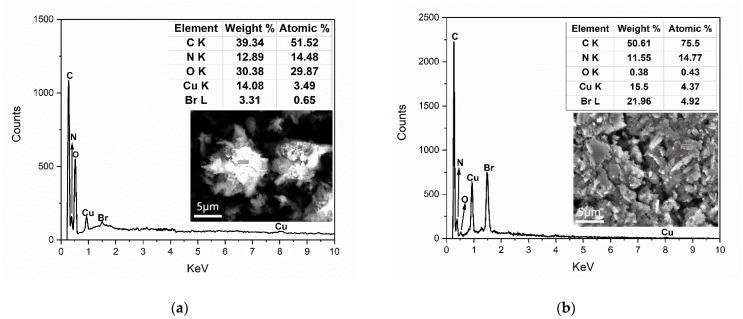
Energy dispersive spectroscopy of (**a**) CP, (**b**) CP-IL. The increase of the mass percentage of C and Br elements indicated that the IL was adsorbed on the CP.

The element composition was detected by an energy spectrometer and detected by the energy spectrum point scanning method (Figure 4). Among them, figure (a) is the energy spectrum line of CP, in which the spectral peaks of the elements were marked. The Scanning Electron Microscope (SEM) diagram shows the selected area of CP sample point scanning, and the table shows the types, mass percentage, and atomic percentage of the main elements in the measured area. Among them, figure (b) is the energy spectrum line of CP IL, in which the spectral peaks of elements were marked. The SEM diagram shows the selected area of CP-IL sample point scanning, and the table shows the types, mass percentage, and atomic percentage of the main elements in the measured area. In the case of little difference in copper content, CP and CP-IL were compared respectively. For the CP, the mass proportion of the C element was 39.34%, while that of the CP-IL was 50.61%. The reason for this phenomenon was the adsorption of ionic liquids: ionic liquids contain a given mass of carbon chain structures. For the Br element, the mass proportion of bromine in the CP was 3.31%, and that in the CP-IL was 21.96%. Br in CP was due to impurities, and the increase of Br in CP-IL was due to the existence of Br in IL In conclusion, these results suggest that IL successfully modified CP particles.

### 3.2. Fourier Transform Infrared Spectroscopy (FT-IR)

**Figure 5:**
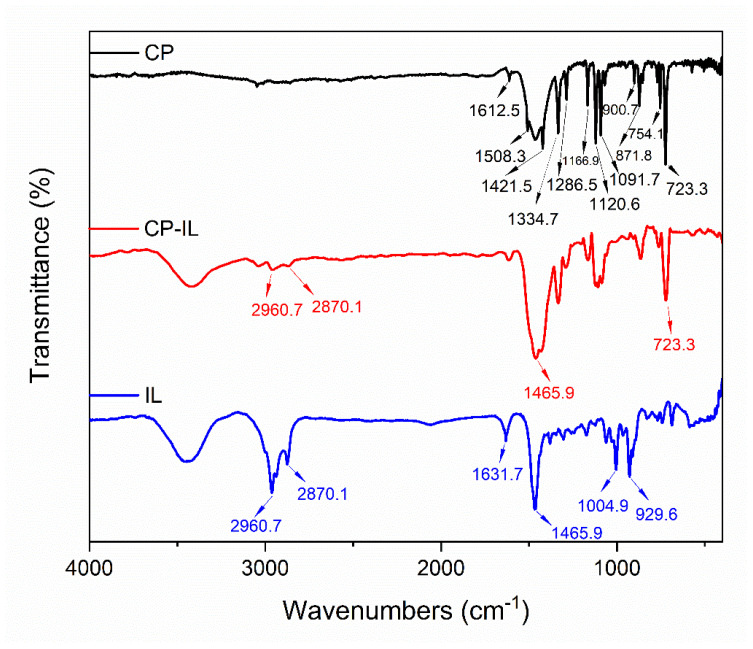
Fourier transform infrared (FT-IR) spectra of CP, CP-IL, and IL.

The chemical functional groups of the CP before modification, CP-IL, and IL were characterized by FT-IR spectroscopy (Figure 5). The FT-IR spectrum of the CP displayed the characteristic peaks at 1612.5, 1508.3, 1421.5, 1334.7, 1286.5, and 1091.7 cm^−1^, which were the stretching vibration bands of plane C-C or C-N of phthalocyanine ring. The in-plane bending vibration bands of C-H in the benzene ring were observed at 1166.9 and 1120.6 cm^−1^, and the out-of-plane bending vibration bands of it at 871.8, 754.1, and 723.3 cm^−1^. The stretching vibration peak of Cu-N was observed at 900.7 cm^−1^. At the same time, the out-of-plane bending vibration bands of the benzene ring also appeared in the range of 400–700 cm^−1^. The FT-IR spectrum of the IL displayed the characteristic peaks at 2960.7 and 2870.1 cm^−1^, which were the stretching vibration peaks of C-H in alkyls or tetrahydropyrrole. The plane bending vibration peak of methyl and methylene was observed at 1465.9 cm^−1^, and the out-of-plane bending vibration peak of C-H in alkyls or tetrahydropyrrole was observed at 1004.9 cm^−1^. The stretching vibration peak of the tetrahydropyrrole ring skeleton and stretching vibration peak of C-N was observed at 1631.7 cm^−1^ and 929.6 cm^−1^ respectively, and Br^−^ had no absorption peak. By combining and comparing the above three infrared spectra [63], it can be found that in the infrared spectrum of CP-IL, there are not only the characteristic peaks of CP but also the characteristic peaks of IL.

### 3.3. Thermogravimetric Analysis (TGA)

**Figure 6:**
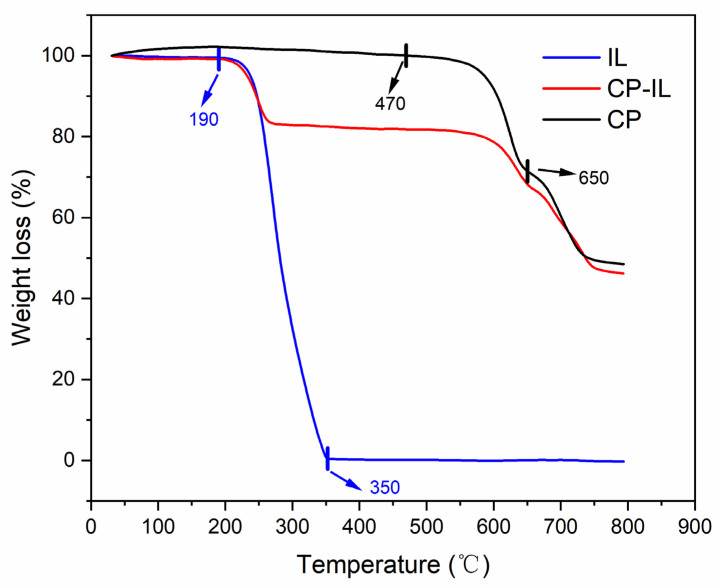
Thermogravimetric analysis (TGA) of CP, CP-IL, and IL.

The CP, CP-IL, and IL were tested with a thermal analyzer under the protection of high-purity nitrogen at a heating rate of 5 °C/min and in the temperature range of 30–800 °C. From the TGA curve (Figure 6), we can see that the weight loss of copper phthalocyanine mainly showed two stages, 470–650 °C, and 650–790 °C. In the range of 470–650 °C, the weight loss of CP was about 28%, which was mainly due to the sublimation, polymerization, dehydrogenation, and denitrification of phthalocyanine ring, and the cyclization of groups around the center Cu-N_4_. In the temperature range of 650–790 °C, CP lost about 23% of its weight. In this temperature range, the central Cu-N_4_ structure was destroyed, the nitrogen atoms were gradually pyrolyzed and separated, and the metal Cu was gradually separated from the central Cu-N_4_ structure, forming metal agglomeration. CP molecule had a planar conjugate macro structure Π, with uniform electron density distribution and high stability. The weight loss of IL was mainly in the range of 190–350 °C, which was mainly due to the ring breaking of pyrrolidine and alkanes in IL. The weight loss of CP-IL in this temperature range was also caused by this reason. The results indicated that IL was coated on the CP.

### 3.4. Particle Size and Zeta Potential

As mentioned earlier, electrophoretic particles play a role in image color rendering in EPDs. Its dispersion stability and electrophoretic mobility in the dispersion medium directly determine the relevant properties of EPDs. Zeta potential is an important factor affecting the stability of the colloidal system. The higher the Zeta potential (positive or negative), the more stable the system. Helmholz-Smoluchowski equation [64] studied the effect of particle surface Zeta potential on particle motion, and gave the formula of particle migration velocity in the dispersion system (Equation (1)):

(1) $$\mu =\frac{\varepsilon \delta }{4\pi \eta },$$

Among them: μ is electrophoretic mobility, 10^−8^ m^2^ v^−1^ s^−1^; *ε* is dielectric constant; *δ* is Zeta potential; *η* is medium electrodynamic viscosity. When the applied voltage was low, the migration speed of electrophoretic particles in a certain suspension was mainly controlled by the Zeta potential on the surface, that is, the amount of charge on the particle surface. In a word, the Zeta potential of particles is caused by the adsorption of relevant ions on the surface. Therefore, the modification of electrophoretic particles was mainly to improve the Zeta potential and further improve the electrophoretic mobility. The Zeta potential test results show that 1-Butyl-1-methylpyrrolidinium bromide has an obvious effect on improving the Zeta potential of Copper (II) phthalocyanine (Table 1). According to Equation (1), the electrophoretic mobility of the CP-IL is 1.04 × 10^−9^ m^2^ v^−1^ s^−1^.

The study used 1-Butyl-1-methylpyrrolidinium bromide mono ionic liquid, which was compared with the 1-Butyl-3-methylimidazolium bromide mono ionic liquid used by Eshkalak [35] et al. (Table 2). The anions in both ionic liquids are Br ^−^, and the lengths of the cationic side chains are also the same. Comparing the ionization ability of the two is to compare the stability of the anions and cations of the two. Since both use bromide ions as anions, it is only necessary to compare the stability of the two cations: due to the positive effect of 1-Butyl-3-methylimidazolium bromide. The charge is located on the carbon-nitrogen double bond, and the double bond is not easy to disperse the positive charge. From the resonance structure, there will be a resonance structure with charge separation above, so the positive ion cannot exist stably. The nitrogen atom of 1-Butyl-1-methylpyrrolidinium bromide does not form a conjugated system, it is connected to an alkyl chain, which has a strong hyperconjugation effect and can well stabilize the positive charge. Therefore, 1-Butyl-1-methylpyrrolidine cation is more stable than 1-Butyl-3-methylimidazole cation, that is, 1-Butyl-1-methylpyrrolidinium bromide is easier to ionize than 1-Butyl-3-methylimidazolium bromide. In conclusion, the modification effect of 1-Butyl-1-methylpyrrolidinium bromide is better than that of 1-Butyl-3-methylimidazolium bromide.

### 3.5. Stability of Electrophoretic Dispersion

**Figure 7:**
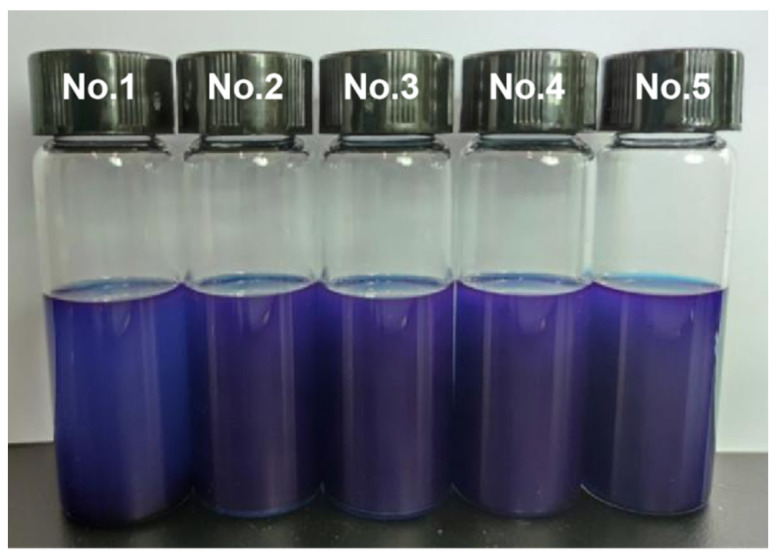
The electrophoretic dispersion was placed for 20 days (No. 1), 15 days (No. 2), 10 days (No. 3), 5 days (No. 4), and 0 days (No. 5).

Meanwhile, to intuitively gauge the stability of the electrophoretic dispersion, sample No. 1 was prepared according to the method of 2.3 on day 0, sample No. 2 was prepared on day 5, and sample No. 3 was prepared on day 10. By analogy, sample No. 5 was prepared on day 20. On the 20th day, five samples were photographed and recorded (Figure 7). The results showed that the electrophoretic dispersion began to settle after standing for 15 days, but the results were not obvious by naked eye observation. The electrophoretic dispersion showed obvious sedimentation after 20 days. In a word, the stability of the electrophoretic dispersion was good.

### 3.6. Display, Luminance, and Chromaticity Coordinate of an EPD Cell

**Figure 8:**
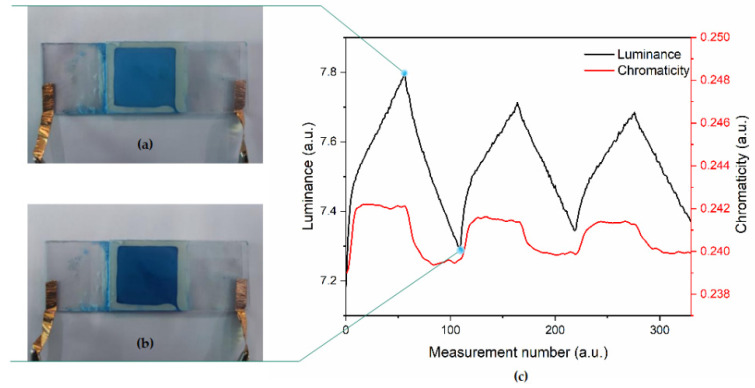
Photograph (top view) of the blue and white dual-color EPD cell with +50 V on the lower plate (**a**). Photograph (top view) of the blue and white dual-color EPD cell with +50 V on the upper plate (**b**). Curve change relationship between luminance and chromaticity coordinate of the EPD in driving process (**c**).

The EPD cell was taken as the measured object, the measurement interval of the colorimeter was 0.17 s, and the chromaticity coordinates of the reference white point were set to (0.3127, 0.3291). During the test, a square wave with a period of 10 s and a voltage of 5 V was generated by the function generator, which was amplified ten times by the voltage amplifier and connected to the EPD cell. Data was collected by the CIE Yxy with a colorimeter. Finally, the data was transmitted to the computer and recorded in real-time with the Admesy software. Three cycles were tested in this work (Figure 8c). The black curve was the luminance of the EPD and the red curve was the chromaticity y coordinate. When +50 V voltage was applied to the upper plate, the white negatively charged particles were driven upward. The luminance of the display increased, the y increased at the same time, and driven towards the light blue direction (Figure 8a); On the contrary, the blue positively charged particles were driven upward, the luminance of the display decreased, and the chromaticity coordinates driven towards the dark blue direction (Figure 8b). However, when the test of the second cycle started, it was found that the brightness of the EPD could no longer reach the brightness of the first cycle. The reason here was that some electrophoretic particles were attached to the surface of the upper plate, resulting in insufficient reflected light received by the colorimeter, but this performance is not obvious in the third cycle.

## 4. Conclusions

A new blue electrophoretic particle for EPD was prepared. The particles were characterized by infrared spectroscopy, energy spectrum analysis, and thermogravimetry. The results showed that 1-Butyl-1-methylpyrrolidinium bromide mono ionic liquid was introduced into the surface of organic pigment Copper (II) phthalocyanine. The particle size was about 1100 nm. The particles had great stability and positive charge in the electrophoretic dispersion. Due to the existence of ionic liquids, the Zeta potential of pigment particles increased to 49.91 mV. The stability experiment result shows that the particle could be stable for at least 20 days. At the same time, the prepared positively charged blue electrophoretic particles and negatively charged white titanium dioxide particles were compounded into blue and white dual-color dispersion, which were encapsulated in an EPD cell. The blue Copper (II) phthalocyanine pigment particles had a reversible electrical response in the EPD cell with a bipolar voltage of ±50 V. This method has been proved to be promising for the preparation of color electrophoretic particles and has potential application prospects in the field of material technology such as EPD. At the same time, the results show that pyrrolidine ionic liquid can be used as a substitute for some surfactants and effective charge control agents.

## Figures and Tables

**Table 1:** The particle size and Zeta potential of CP, and CP-IL.

Sample	Particle Size (nm)	Zeta Potential (mV)
CP	932.90	32.42
CP-IL	1138.37	49.91

**Table 2:** Comparison of structures and properties of two ionic liquids.

Ionic Liquids	Chemical Constitution	Side Chain Length	Zeta Potential (mV)	Electrophoretic Mobility (m^2^ v^−1^ s^−1^)
1-Butyl-3-methylimidazolium bromide	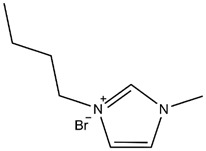	4C	41.60	9 × 10^−10^
1-Butyl-1-methylpyrrolidinium bromide	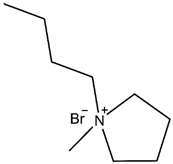	4C	49.91	1.04 × 10^−9^
